# Artificial Intelligence in Epigenetic Studies: Shedding Light on Rare Diseases

**DOI:** 10.3389/fmolb.2021.648012

**Published:** 2021-05-05

**Authors:** Sandra Brasil, Cátia José Neves, Tatiana Rijoff, Marta Falcão, Gonçalo Valadão, Paula A. Videira, Vanessa dos Reis Ferreira

**Affiliations:** ^1^Portuguese Association for CDG, Lisbon, Portugal; ^2^CDG & Allies – Professionals and Patient Associations International Network (CDG & Allies – PPAIN), Caparica, Portugal; ^3^UCIBIO, Departamento de Ciências da Vida, Faculdade de Ciências e Tecnologia, Universidade NOVA de Lisboa, Lisbon, Portugal; ^4^Instituto de Telecomunicações, Lisbon, Portugal; ^5^Departamento de Ciências e Tecnologias, Autónoma Techlab — Universidade Autónoma de Lisboa, Lisbon, Portugal; ^6^Electronics, Telecommunications and Computers Engineering Department, Instituto Superior de Engenharia de Lisboa, Lisbon, Portugal

**Keywords:** epigenetics, epigenomic, artificial intelligence, machine learning, personalized medicine, rare diseases (RD)

## Abstract

More than 7,000 rare diseases (RDs) exist worldwide, affecting approximately 350 million people, out of which only 5% have treatment. The development of novel genome sequencing techniques has accelerated the discovery and diagnosis in RDs. However, most patients remain undiagnosed. Epigenetics has emerged as a promise for diagnosis and therapies in common disorders (e.g., cancer) with several epimarkers and epidrugs already approved and used in clinical practice. Hence, it may also become an opportunity to uncover new disease mechanisms and therapeutic targets in RDs. In this “big data” age, the amount of information generated, collected, and managed in (bio)medicine is increasing, leading to the need for its rapid and efficient collection, analysis, and characterization. Artificial intelligence (AI), particularly deep learning, is already being successfully applied to analyze genomic information in basic research, diagnosis, and drug discovery and is gaining momentum in the epigenetic field. The application of deep learning to epigenomic studies in RDs could significantly boost discovery and therapy development. This review aims to collect and summarize the application of AI tools in the epigenomic field of RDs. The lower number of studies found, specific for RDs, indicate that this is a field open to expansion, following the results obtained for other more common disorders.

## Introduction

To date, more than 7,000 rare diseases (RDs) have been described, collectively affecting about 350 million people globally^1^ (Ronicke et al., 2019). Approximately 80% of RDs have a genetic origin and about 75% affect children (Ekins, 2017). Most RDs are monogenic (Mendelian) and for that, are considered “simple” traits. However, RDs are now more and more considered complex traits due to: (a) phenotypic and genetic heterogeneity, (b) complex mutation spectrum (e.g., existence of modifier genes), and (c) unknown gene-disease associations and genetic mechanisms. They face the problem of “missing heritability,” which impairs discovery, diagnosis, and patient care (Scriver and Waters, 1999; Berdasco and Esteller, 2019; Maroilley and Tarailo-Graovac, 2019).

Epigenetics is the mechanism by which changes in gene expression occur without changing the DNA sequence. It is the product of a complex interaction between the genotype of an individual and the surrounding environment and plays a determinant role in disease development and progression (Romanowska and Joshi, 2019; Rauschert et al., 2020). Epigenetics includes DNA methylation, histone post-translational modifications and variants, regulation by small non-coding RNAs (sncRNAs) (e.g., RNA interference and microRNAs), and nuclear organization, which are responsible for appropriate activation or repression of genes (García-Giménez et al., 2012; Wen and Tang, 2018). Such processes represent a link to the lifestyle and environmental contributions and can be detected at early stages of the disease and in all genomic contexts not only in coding regions but also in non-coding regions (García-Giménez et al., 2012). Hence, epigenetic biomarkers represent an attractive option in clinical research and practice. Epigenetic modifications are technically stable, particularly DNA methylation, thus facilitating their identification. They are also quite stable in fluids and tissues that are commonly accessed in research and clinical practice. Increasing efforts are being made to develop new methodologies (e.g., single cell epigenome sequencing techniques) and tests to implement epigenetic biomarkers and their monitoring in clinical practice (Wen and Tang, 2018). In fact, clinical epigenetics is already established in Oncology with biomarkers approved by the US Food and Drug Administration (FDA) for diagnosis, prognosis, or therapy response, as well as epigenetic-based therapies. It is also becoming a growing field in neurological, immunological, metabolic, and infectious diseases (Berdasco and Esteller, 2019; Rauschert et al., 2020).

The development of personalized medicine is tightly connected to the selection, analysis, and integration of information from different “omics” approaches as well as patient and medical data (Rauschert et al., 2020). In this “big data” context, artificial intelligence (AI), particularly machine learning (ML), the area of AI that develops tools “that can be used to design and train algorithms to learn from and act on data” (Toh et al., 2019), can have a significant role in assisting researchers and clinicians in integrating, interpreting, and managing large and complex data sets (Rauschert et al., 2020).

Machine learning algorithms can be roughly classified as: (a) supervised learning, (b) unsupervised learning, and (c) reinforced learning.

In supervised learning, the algorithm is given both the input data and the corresponding target data, uncovering the relationship between the input and target data. Classification and regression tasks are examples of supervised learning.

In unsupervised learning, only input data is given to the algorithm, which then has to identify the existing underlying structure. Clustering (the automatic assignment of object groups into clusters/groups) and density estimation are examples of unsupervised learning.

Finally, in reinforcement learning, the goal of the algorithm is to find the most suitable action in order to maximize a reward, which, in turn, depends on the action (Brasil et al., 2019).

In ML tools, independent variables are designed as *p*, while the sample size is denoted by *n*. Most statistics-based ML approaches require a high amount of structured data (*p*) from a large sample set (*n*) to train the model, so it can be able to make true and reliable inferences (Ma and Zhang, 2019). In RDs, the high number and variety of data obtained from different “omics” allied to a reduced sample size (“big *p*, small *n*” problem) can hinder the application of AI tools in RDs (Mei and Wang, 2016; Ma and Zhang, 2019). Adaptation and modification of current AI/ML tools and the generation of new and more flexible tools are needed to fully explore multi “omics” data. Despite these difficulties, AI/ML tools have been successfully applied in RDs (Brasil et al., 2019). AI (particularly ML) allied to epigenomics, has been used to diagnose or classify several disorders (e.g., cancer, cerebral palsy, and neurodevelopmental syndromes) (Rauschert et al., 2020). Genetic mutations in genes related to DNA methylation or in histone modifiers were found in Rett syndrome, hereditary sensory autonomic neuropathy type 1E, and Cornelia de Lange syndrome, among other RDs. Also, errors in the imprinting process (a process regulated by DNA methylation and histone modifications) are critical in Angelman, Prader–Willi, and Beckwith–Wiedemann syndromes. Thus the disruption of the epigenome and its association with RDs, indicates that the interplay between genetics and epigenetics should be considered when addressing the etiology of RDs (Nguyen, 2019).

In order to assess the state-of-the-art of the use of AI in epigenomic studies in RDs, we performed a literature revision, having collected and structured the information regarding their application for: (a) diagnosis, (b) disease characterization, and (c) therapeutic approaches in RDs.

This review gathers AI-based tools for epigenomic studies for biomedical research in RDs, aiming to increase the knowledge and awareness of these applications.

## Materials and Methods

For this review, we defined a set of keywords related to RD, AI, epigenetics, and Tools. Then, we adapted our custom Python script and prepared the input file (Brasil et al., 2019) to combine keywords from three first groups (triple terms) and four groups (quadruple terms) to search in the Medline database, using PubMed as the search engine through its application programming interface (API), the Entrez Programming Utilities (Sayers, 2010; Supplementary Figure 1). To use that API, we used libraries from the Biopython project (Cock et al., 2009; Supplementary Note 1 and Supplementary Table 1). This script limited the results for each of the keyword combinations to the thousand most relevant articles. It also eliminated duplicate entries and retrieved the correspondent Medline data (Title, Abstract, and MeSH terms) from each article. Then, we developed a custom Python script that extracts information to LaTeX from the output of the previous script and generates a PDF to each article with that information (e.g., title, authors, date, abstract, mesh terms, Source, PubMed Unique Identifier, and PubMed Central Identifier; Supplementary Note 2).

Three rounds of manuscript selection were performed, each one with different selection criteria:(1) Articles were selected based on title and abstract reading by two researchers; (2) Articles matching the selection criteria were included for the second round of full-manuscript reading by five researchers; (3) A final round was performed by an independent researcher, who analyzed the AI tools/algorithms to guarantee uniform selection criteria (Supplementary Figure 2).

Inclusion criteria were as follows:

(1) English-written articles that included the title, abstract, and MeSH terms;
(2) Articles that combined AI algorithms (or families of algorithms) with epigenomics to address specific problems related to RDs;
(3) RDs with Orpha codes (from Orphanet classification);

Reviews were excluded from the results and only used in the introduction or discussion for contextualization purposes. To guarantee that, we have not missed relevant articles, we screened the references from the included reviews.

## Epigenetics and AI in RDs: Existing Literature

**FIGURE 1:**
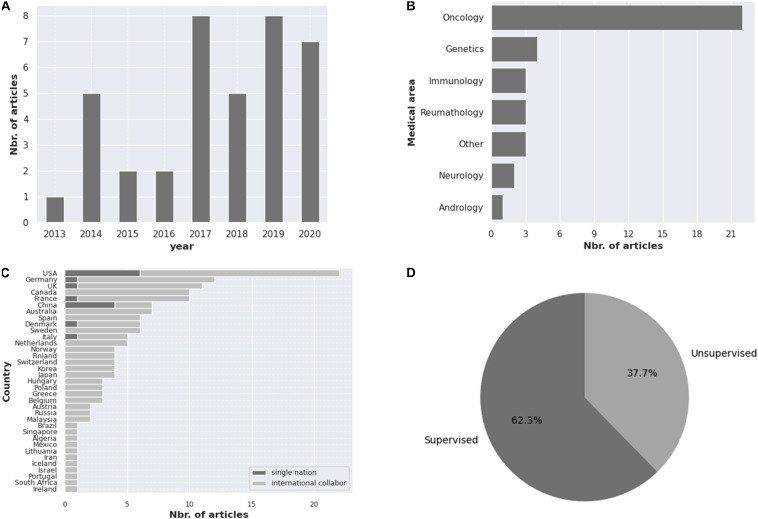
Compilation of the information obtained in this review regarding the **(A)** number of articles published from 2013 to 2020, **(B)** the medical areas covered, **(C)** the number of publications per country, highlighting research collaboration and **(D)** percentage of unsupervised and supervised AI tools reported.

Our search revealed 38 studies using AI tools for epigenetic studies in RDs. Over the 7-year time period considered in this review, publication numbers increased from 1 in 2013 to 7 in 2020, with the highest number of publications in 2017 (Figure 1A). There was a great heterogeneity among the different tools used, the disorders reported, and the size of the samples as well as for the epigenomic data used. Most studies were related to rare cancers (*n* = 22) (Figure 1B), highlighting the importance that epigenetics has in cancer studies, followed by Mendelian disorders (*n* = 4). Studies were developed in different countries, with the largest number of publications originating from the United States (*n* = 22) (Figure 1C). Both unsupervised and supervised leaning methods were reported (Figure 1D). Among the supervised methods, we found support vector machine (SVM) (*n* = 4), elastic net method (*n* = 3), linear regression (*n* = 1) as the major tools identified. In the unsupervised methods, hierarchical clustering (*n* = 9) was the most utilized. A list of AI tools used in epigenetic analyses in RDs is compiled in Table 1. The majority of tools identified were supervised, and amongst them, PLINK, a tool based on a linear regression model and used for genotype/phenotype data analysis was the most described. DeepTools, based on k-means clustering was the only unsupervised ML tool described (Table 1).

**TABLE 1:** List of available AI and ML-based tools used for epigenetic studies in RDs.

**Function**	**References**	**Software/Platform/Algorithm**	**AI/ML method**	**Disease(s)**	**Classification**
Annotates and prioritizes non-coding regulatory variants	Fu et al., 2014	FunSeq2 http://funseq2.gersteinlab.org/	Scoring scheme, using conservation, regulatory, and other measures	Medulloblastoma	Supervised/Unsupervised
Discover variants associated to specific Mendelian disorders	Smedley et al., 2016	Genomiser https://hpo.jax.org/app/tools/genomiser	ReMM framework/RF classifier	Beckwith-Wiedemann syndrome (ORPHA:116), beta thalassemia (ORPHA:848), Marie Unna hereditary hypotrichosis (ORPHA:444)	Supervised
Causal variant analysis and identification	Farh et al., 2015	PICS	Bayesian approaches	Immune disorders	Supervised
Predict the effect of regulatory variation	Vuckovic et al., 2020	Delta SVM http://www.beerlab.org/deltasvm/	SVM classifier	Blood cell traits	Supervised
Genes and gene sets prediction	Hou et al., 2017	GeneMANIA https://genemania.org/	Fast heuristic algorithm derived from ridge regression	RVF	Supervised/Unsupervised
miRNA target prediction and functional annotation		miRDB	MirTarget		
Detect statistically significant interaction events in Capture HiC data	McMaster et al., 2018	CHiCAGO (http://regulatorygenomicsgroup.org/chicago)	Convolution background model	Waldenstrom macroglobulinemia	Supervised
Identifies the precise location of active TREs	Chu et al., 2018	dREG.HDhttps://github.com/Danko-Lab/dREG.HD	Epsilon SVR with a Gaussian kernel	Human glioblastoma	Supervised
Genotype/phenotype data analysis	Luzón-Toro, 2015; Glubb et al., 2017; Vijayakrishnan et al., 2017; Moreno-Moral et al., 2018; Cochran et al., 2020	PLINK (https://zzz.bwh.harvard.edu/plink/)	Linear regression model	EOC, sMTC and PTC, leukemia	Supervised
miRNA-disease associations	Liu et al., 2019	NBMDA	Gaussian interaction profile kernel similarity/KNN	Esophageal, breast, and colon neoplasms	Supervised
Learning and characterization of chromatin states	Bien et al., 2017	ChromHMM http://compbio.mit.edu/ChromHMM/	HMM	CRC	Supervised
Analysis of high-throughput sequencing data (ChIP-seq, RNA-seq, MNase-seq)	Han et al., 2016	DeepTools https://deeptools.readthedocs.io/en/develop/	*k*-means clustering	AML	Unsupervised

*AML, acute myeloid leukemia; CRC, colorectal cancer; eQTL, expression quantitative trait loci; EOC, epithelial ovarian cancer; HMM, Hidden Markov Model; NB, negative binomial; KNN, *k*-nearest neighborhood, PICS, Probabilistic identification of causal SNPs; PTC, papillary thyroid carcinoma; RF, Random forest; ReMM, Regulatory Mendelian mutation; RVF, Rift valley fever; sMTC, sporadic medullar thyroid carcinoma; SVM, support-vector machine; SVR, support-vector regression.*

### Unsupervised ML Algorithms

Clustering is the separation of a set of data into different groups (clusters) according to their similarity (i.e., data with similar characteristics is grouped in the same cluster and data with different clusters that are not similar), which is measured in the distance (e.g., Euclidian distance) (Omran et al., 2007; Park and Jun, 2009). Clustering can be divided into hierarchical, in which the clusters are divided in a cluster tree with each cluster containing a part of the data set, and partitional clustering (PC), in which the data set is divided into a specific number of clusters (Omran et al., 2007). Hierarchical clustering (HC) algorithms are independent of the initial conditions and they do not need an initial definition of the number of clusters; however, they are not suitable for large data sets, and do not allow for pattern flexibility (i.e., data assigned to a cluster cannot be moved to another) and may not be able to differentiate among overlapping clusters. In order to circumvent these disadvantages, PC can be used (Omran et al., 2007). PC encompasses k-means clustering, in which data is organized in several (k) different clusters based on their similarity with the mean value of each particular cluster in its center (Park and Jun, 2009; Dey, 2016). K-means clustering is simple and fast, allowing its use on large datasets; however, since the results depend on the initial random assignments, results are not consistent and may vary with each run. Furthermore, it is necessary to define a mean value, which is not always possible and it is also sensitive to outliers. In these cases, the application of k-medoids variants is an alternative (Singh et al., 2011). Partitioning around medoids (PAM) is the most powerful among the many k-medoids algorithms; however, due to its time complexity, it does not work well in large data sets (Park and Jun, 2009).

Validation of cluster results is fundamental in cluster data analysis. Simulated perturbations of the original data set can be used to infer clustering results stability with respect to sampling variability. This is known as resampling and can be used for cluster result validation (Monti et al., 2003). Consensus clustering is used when a given number of clusters have been generated for a determined dataset and it is necessary to find a unique clustering which is the best fit to the existing set of clusters (Li et al., 2007). It is a resampling-based method used to find consensus within multiple runs of clustering algorithms; it assesses the number of clusters that exist within the data set and their stability. It can also express the consensus over several runs of random start clustering algorithms, such as k-means (Monti et al., 2003). Non-negative matrix factorization (NMF)-based consensus clustering can be applied to improve the robustness and performance of clustering algorithms (Li et al., 2007).

Recursively partitioned mixture model (RPMM) (Houseman et al., 2008) is a model-based hierarchical clustering method for high-dimensional data (Koestler et al., 2013). It robustly estimates the number of clusters (k classes) in the data analyzed and is effective in attributing to the relative propensity of the subjects within each predicted class. However, the violation of the assumption of class conditional independence leads to model over-fitting (Koestler et al., 2013).

Gaussian process (GP) model is a non-parametric Bayesian method used for supervised ML that allows for parsimonious temporal inference and the incorporation of prior information into the model. It has been used particularly for gene expression time series analysis (Park and Choi, 2010; Hensman et al., 2013). Hierarchical GPs allow for the clustering of expression data while taking into account, inner cluster variance. The mixture of hierarchical GP (MOHGP) model is based on a hierarchy of GPs to model the mean of the cluster and subsequently de deviation of each time-course within the cluster from that mean^2^.

Multifactor dimensionality reduction (MDR) was developed to detect interactions between genes and/or between genes and environment in small datasets with variables organized into independent categories (Ritchie et al., 2001; Motsinger and Ritchie, 2006). MDR neither assumes particular genetic models nor estimates any parameters (non-parametric) and unlike logistic regression, it can be used for high-dimensional data analysis (Ritchie et al., 2001). Classification and prediction are assessed by cross-validation (CV) and permutation testing (Gola et al., 2016).

Principal component analysis (PCA) is a multivariate statistical technique with multiple applications. Given an observational data table with several dependent variables, in general, inter-correlated PCA is used to extract the most important information (i.e., principal components) and analyzing the structure of both observations and variables, while simplifying data set description (Wold et al., 1987; Abdi and Williams, 2010). In theory, PCA can be applied to any data matrix at the initial steps of multivariate analysis as means of identifying outliers and establish classes. For classification problems, extensions to the PCA algorithm must be used (Wold et al., 1987).

Unsupervised clustering has been used in epigenomics studies in RDs for several purposes that are presented below.

#### Diagnosis: Mutation Detection and/or Prediction

Sorenson et al. (2017) performed a high-resolution comparative genomic hybridization (aCGH) and RNA sequencing (RNA-seq) to analyze chromosomal alterations and dysregulated gene expression in tumor specimens of patients with fibrolamellar hepatocellular carcinoma (FL-HCC, ORPHA:401920). The PAM method was used to perform clustering of RNA-seq data, while the hclust function in R was used to perform hierarchical clustering (with Euclidian distance as similarity measure) of samples and genes. The authors found dysregulation of several gene sets, including genes related to chromatin remodeling (C10orf90), contributing to elucidate the genomic and transcriptomic landscape of this rare disease (Sorenson et al., 2017).

#### Biomarkers and Prognosis

Hierarchical clustering was used to examine genome-wide methylome of uveal melanoma (ORPHA:39044) demonstrating that *RAB31* (a member of the RAS oncogene family) unmethylation is a predictor of poor outcome. Analysis of tumor and blood samples of patients with retinoblastoma (ORPHA:790) uncovered hypermethylation of cathepsin Z (CTSZ), metallothionein 1 H (MT1H) and homeobox C4 (HOXC4) genes as well as hypomethylation of the miR-17-92 (oncomir-1, a potent oncogenic miRNA) cluster, setting a specific methylation signature than can be used for diagnosis and therapeutic avenues (Berdasco et al., 2017).

Koduru et al. (2017) performed hierarchical clustering by means of stringent statistical analysis (*p* < 0.001) on sncRNA sequencing data from 45 adrenocortical carcinoma (ACC, ORPHA:1501), a rare and aggressive type of cancer and 30 adrenocortical adenomas (ACAs), a benign adrenocortical tumor. PartekFlow^®^ software, version 5.0 (Partek, Inc., St. Louis, MO, United States) was used to assemble FASTQ files from small RNA sequencing data to human genome hg19 clustering and allowed the identification of several differentially regulated microRNAs (miRNAs), particularly piwi-interacting RNAs (piRNAs), which have been related to epigenomic modeling; in ACC that could serve as new diagnoses biomarkers as well as new therapeutic targets (Koduru et al., 2017).

Job et al. (2020) used a mining approach of transcriptome data to identify long non-coding RNAs (lncRNAs) specific for PCPGs molecular groups and metastatic progression. ConsensusClusterPlus R package was used to perform unsupervised classification of lncRNAs. Receiver operating characteristic curve (ROC) analyses were used to identify a putative lncRNA that discriminates the benign from metastatic tumors in patients with *SDHx* mutations and is associated with poor clinical outcome of *SDHx* carriers (Job et al., 2020).

In order to provide evidence for future genetic screening guidelines, Waszak et al. (2018) analyzed whole-genome and exome sequences as well as DNA methylation in retrospective and prospective cohorts of patients with medulloblastoma (ORPHA:616). K-means consensus clustering analysis of all CpG probes allowed for the definition of four consensus molecular subgroups. Moreover, rare variant burden analysis revealed a genetic predisposition in at least two of these subgroups. Hence, the authors propose the establishment of genetic counseling and genetic testing as a standard-of-care procedure in these patients (Waszak et al., 2018).

DNA replication timing (RT) is a powerful cell type-specific epigenetic marker with a high intra-cell conservation level that is altered in disease states. Cluster 3.0 was used to perform hierarchical and k-means clustering of RT-variable regions, allowing for the identification of a specific RT signature that discriminates between progeroid syndromes and natural aging in patients with Hutchinson–Gilford progeria syndrome (HGPS, ORPHA:740) and Rothmund–Thomson syndrome (RTS, ORPHA:2909) (Rivera-Mulia et al., 2017). Furthermore, an association between *TP63* RT alterations and the characteristic phenotypic defects of this family of disorders was also established (Rivera-Mulia et al., 2017).

#### Disease Classification/Characterization

Diffuse intrinsic pontine glioma (DIPG, ORPHA:497188) is a cancer of the pediatric pons, characterized by a unique substitution to methionine in histone H3 at lysine 27 (H3K27M). To unveil the pathobiology of DIPG, Nagaraja et al. (2019) performed active chromatin profiling in 25 primary tumor samples and 5 non-malignant pediatric pontine tissue samples, as well as isogenic H3K27M expression in early oligodendrocyte precursor cells (eOPCs). K-means clustering was used for chromatin as well as enhancers and promoters analysis, revealing five states of enhancer and promoter activation. Most samples were separated into three groups: normal pons, H3.1K27M DIPG, and H3.3K27M DIPG, suggesting that H3.3K27M and H3.1K27M DIPG should be considered as functionally distinct subgroups in both preclinical and clinical considerations (Nagaraja et al., 2019).

Epigenetics plays an important role in tissue differentiation and disease modification. However, the role of epigenetics in sexual dimorphisms is not well understood. Ammerpohl et al. (2013) performed microarray-based methylation profiling in genital fibroblasts of 46, XY individuals with androgen receptor (AR) pathway disruption (ORPHA:754). DNA methylation analysis was performed with HumanMethylation27 Bead-Chips and hierarchical cluster analyses based on average beta-values were performed using OMICS Explorer. Results showed that changes in DNA methylation marks in the epigenome by androgen lead to sexual dimorphism programming (Ammerpohl et al., 2013).

Pallister Killian Syndrome (PKS, ORPHA:884) also known as tetrasomy 12p and isochromosome 12p mosaicism is a rare chromosomal aneuploidy with a highly conserved phenotype. Kaur et al. (2014) performed a genome-wide expression analysis in skin fibroblasts of 17 PKS probands, using the Affymetrix Human Genome U133 plus 2.0 arrays. Robust multi-array average (RMA) method was used to normalize and summarize Affymetrix raw data. The normalized data were then analyzed by (PCA. The authors identified 354 differentially expressed genes in PKS probands and evidence for a critical region on 12p13.31. Furthermore, downregulation of *ZFPM2*, *GATA6*, and *SOX9*, and overexpression of *IGFBP2* might be associated with PKS clinical phenotype (Kaur et al., 2014).

Assié et al. (2014) resorted initially to the RPMM, to identify DNA methylation–based ACC clusters, which were associated with poor prognosis or with extensive hypomethylation of CpG sites outward of CpG islands. Then resorting to a consensus clustering tool, they identified clusters, with deregulation of the miRNA expression. The molecular classification of the disease was refined using this work (Assié et al., 2014).

#### Disease Etiology

5-Hydroxymethylcytosine (5hmC) is an intermediate of DNA demethylation as well as a potential epigenetic mediator, modulating an array of biological processes and human diseases. Han et al. (2016) developed a method for 5hmC sequencing which allows genome-wide profiling of 5hmC using a limited amount of genomic DNA. This technology was used to profile leukemia stem cells from a murine model of *Tet2*-mutant acute myeloid leukemia (AML, ORPHA:519) and to obtain high-quality maps of 5hmC in tumor-initiating cells. K-means clustering and calculation of genome-wide correlations were performed with DeepTools, a suite of Python tools for the analysis of high-throughput sequencing data (e.g., ChIP-, RNA-, or MNase-seq). The change of 5hmC patterns in AML is strongly associated with differential gene expression, highlighting the importance of dynamic alterations of 5hmC in transcription regulation in AML. Covalent 5hmC labeling offers an efficient approach to detect and study DNA methylation dynamics in *in vivo* disease models and in limited clinical samples (Han et al., 2016).

### Supervised ML Algorithms

Linear regression predicts continuous dependent variables from other given independent variables (Altman and Krzywinski, 2015). In the presence of categorical dependent variables (e.g., biomedical data), logistic regression can predict both variable value and associated probability (Lever et al., 2016). Both linear and logistic regression models are powerful tools for the classification and class probability prediction. However, the presence of correlation over multiple predictors is difficult in coefficient interpretation (Lever et al., 2016).

To optimize the performance of the logistic regression model in the presence of a high number of variables, the imposition of penalties (regularization) can be performed. There are three main penalized regression models: (i) ridge regression in which the coefficients of variables with minor contributions are set close to zero, without eliminating any variables. This is useful when all the variables need to be incorporated in the model; (ii) the least absolute shrinkage and selection operator (LASSO) regression that uses the penalty of the sum of the absolute values of its components (ℓ_1_-norm) (Vidyasagar, 2015), in which the coefficients of the minor variables are set to be exactly zero and the less significant variables are eliminated from the model; and (iii) elastic net regression which is a combination of the previous two (some coefficients are set to be exactly zero, while others are only approximately zero) (Li and Sillanpää, 2012)^3^.

Partial least squares regression (PLSR) infers relationships between two sets of observed variables that have latent variables within and can be used to solve both single- and multi-label problems (Chen et al., 2019). PLSR is a good choice for prediction due to its computational efficiency, simplicity, and dimensionality reduction (Chen et al., 2019).

Classification and regression trees (CART) used combinations of explanatory variables that may be categorical (classification) and/or numeric (regression) to repeatedly split the data into more homogeneous groups and are suited for the analysis of complex and unbalanced data. CART is easy to interpret, flexible, and able to handle variable data sets and to handle missing values in response and/or explanatory variables (De’ath and Fabricius, 2000).

*k*-Nearest Neighbor (*k*-NN) classification is based on two steps: (i) identification and determination of the nearest neighbors and (ii) class determination using the set of neighbors and is a simple and easy method for classification. However, it can have a low run-time performance for large training data sets and it is highly sensitive to redundant features. Finally, this method is outperformed by more powerful tools, such as support vector machines (Cunningham and Delany, 2007).

Hidden Markov model (HMM) is a probabilistic model based on the assumption that the sequence of the observed data arises from some sequence of underlying hidden discrete states and is widely used for sequence analysis (Bousquet et al., 2004; Ben-Hur et al., 2008). HMM can be applied directly to raw data and can handle inputs of variable length; however small data sets can lead to over-fitting. The over-fitting problem can be solved by the use of hierarchical or factoral HMMs (Degirmenci, 2014).

Support vector machine algorithms are used mostly for classification, and classification is based on the definition of the best hyper-plane to separate all data points in one class from the other classes. Separation can be made using linear and/or non-linear boundaries. For non-linear classification problems, a kernel function must be applied (Dey, 2016; Savas and Dovis, 2019). A Gaussian kernel has the shape of a Gaussian curve and is used for smoothing (i.e., noise reduction). Support vector regression (SVR) algorithms are used for regression and can be considered an extension of SVR; however, SVR has a more flexible tolerance for error (Awad and Khanna, 2015).

Bayesian networks are direct acyclic graph representations of random variables and their conditional probability based on Bayes’ theorem to create decision trees. Bayesian networks are robust against missing data and avoid overfitting, but the network structure can be difficult to interpret and they do not perform well in the presence of many features (Ehsani-Moghaddam et al., 2018).

Rain forest (RF) method consists of a set of decision trees in which each tree provides a classification for the input data and the final classification is obtained by the most voted prediction (Chen et al., 2014). Boruta method is an algorithm that copes with RF problems by adding more randomness to the system by making a randomized copy of the system, merging it with the original, and building a classifier for the extended system (Kursa et al., 2010).

Machine learning algorithms do not perform well with the imbalanced dataset (classes are not relatively represented). For imbalanced data, the performance of ML algorithms cannot be correctly assessed (Chawla et al., 2002). To overcome class imbalance, re-sampling of the original dataset (over-sampling of the minority class and/or under-sampling of the majority class) can be applied. Synthetic minority over-sampling technique (SMOTE) is “an over-sampling approach in which the minority class is over-sampled by creating ‘synthetic’ examples rather than by over-sampling with replacement.” Hence, it improves the classifier accuracy for minority classes (Chawla et al., 2002).

#### Diagnosis: Mutation Detection and/or Prediction

The correct identification of genes and mutations is essential for diagnosis and disease prediction. The identification of drivers (mutations that lead to oncogenesis) has focused mainly on genome coding regions. However, driver events can also be caused by mutations affecting regulatory elements. FunSeq2, a tool that combines a small-scale informative data context generated from large-scale resources (e.g., ENCODE data) and a variant prioritization pipeline was developed by Fu et al. (2014) to annotate and prioritize somatic variants (alterations in DNA that occur in any body cell, besides germ cells, after conception^4^), particularly regulatory non-coding mutations. The authors have correlated epigenetic modifications with gene expression levels across 20 different tissues and established associations among all non-coding variants in the regulatory elements and potential target genes. Furthermore, FunSeq2 allows user data input on regions or chromatin marks, allowing for the identification of novel correlations between coding genes and regulatory elements (Fu et al., 2014).

Farh et al. (2015) combined genetic and epigenetic fine mapping to identify causal variants in autoimmune disease-associated loci and infer their functions. The authors developed Probabilistic Identification of Causal SNPs (PICS), an algorithm based on Bayesian approaches, to estimate, in 21 autoimmune diseases, the probability that an individual single-nucleotide polymorphism (SNP) is a causal variant taking into account the haplotype structure and observed pattern of association at the locus. Through PICS, the authors identified that about 90% of causal variants are non-coding, with 60% mapping to immune-cell enhancers and gained histone acetylation (Farh et al., 2015).

McMaster et al. (2018) used CHiCAGO, a convolution background model, to analyze significant chromatin interactions between patients with Waldenström macroglobulinemia (WM, ORPHA:33226) and controls in a two-stage GWAS. They identified two high-risk non-coding SNPs, rs116446171 and rs117410836 at 6p25.3 and 14q32.13, respectively. Rs116446171 is located near *IRF4*, *DUSP22*, and *EXOC2*, which are implicated in a variety of lymphoid cancers and might represent an important non-coding variant for WM risk (McMaster et al., 2018).

Crippa et al. (2014) used a probabilistic HMM applied to human embryonic stem cell (HMES) to identify putative regulatory sequences in ChIP-seq data of a patient with trichorhinophalangeal syndrome (TRPS, ORPHA:324764), a complex autosomal dominant malformative disorder, characterized by distinctive craniofacial and skeletal abnormalities.

Myotonic dystrophy type 1 (DM1, ORPHA:206647) is a multisystem disorder that affects skeletal and smooth muscle as well as the central nervous system. It is caused by a CTG repeat expansion. Longer CTG expansions are associated with greater symptom severity and earlier age at onset. To directly quantify the treatment effect by the reduction of the CTG repeat, Kurkiewicz et al. (2020) developed a model based on partial least squares regression (PLSR), that is able to predict the size of the DM1CTG repeat and the effect that has on mRNA expression.

Interferon-induced transmembrane protein 5 (IFITM5) is a positive modulator of bone mineralization. However, little is known about its regulation. Mo et al. (2014) performed a predictive search of miRNAs targeting *IFITM5* in human osteosarcoma (ORPHA:668) cell lines using DIANA-microT, a tool based on the microT algorithm, which is particularly trained on a positive and a negative set of miRNA recognition elements (MREs) located in both the 3′-UTR and CDS regions. The authors identified miR-762 as a novel regulator of *IFITM5*, shedding new light on the roles of miRNAs in osteoblast differentiation (Mo et al., 2014).

Rare *de novo* epi-variants, a class of genetic variants involving changes in DNA methylation patterns of a reduced number of CpGs at a particular locus, are found at a higher frequency in subjects presenting neurodevelopmental syndromes with or without congenital anomalies (ND/CA). This leads to the hypothesis that some of these epi-variants may contribute to the pathogenesis of some unexplained ND/CAs (Aref-Eshghi et al., 2019). A multiclass SVM with a linear kernel classification model was developed to analyze genome-wide DNA methylation data leading to the detection of an epi-signature associated with14 ND/CA syndromes. The model allowed for the definitive diagnosis and classification of several patients from a large cohort of 965 ND/CA-affected subjects with no previous diagnostic, as well as an additional cohort of 67 subjects with uncertain clinical diagnosis (Aref-Eshghi et al., 2019).

Early onset of Alzheimer’s disease (EOAD, ORPHA:1020) and frontotemporal dementia (FTD, ORPHA:282) exhibit heritability patterns that cannot be explained by currently known genetic contributors, suggesting additional genetic factors contributing to the disease. Cochran et al. (2020) analyzed variant associations between EOAD and FTD vs. controls. Variant annotation and predicted deleteriousness were obtained with CADD, a tool that uses a machine learning model trained on a binary distinction between simulated *de novo* variants and variants that have arisen and become fixed in human populations. PLINK was used to assess single common variant contributions from GWAS data. This analysis identified *TET2*, which promotes DNA de-methylation, as a risk component for multiple neurodegenerative disorders, such as EOAD and FTD (Cochran et al., 2020).

Coffin–Siris syndrome (CSS) is an extremely rare syndrome associated with intellectual disability. Pranckėnienė et al. (2019) reported a novel *de novo* splice site variant detected by whole exome sequencing (WES) in the *ARID1B*, responsible for CSS. Potential variants in the ARID1B protein were assessed with Pfam 32.0 database, which is a large collection of protein families, each represented by multiple sequence alignments and HMMs^5^. The *de novo* variant is responsible for a truncated protein, resulting in the loss of the BAF250 domain. This domain is part of the SWI/SNF−like ATP−dependent chromatin remodeling complex, which regulates gene expression (Pranckėnienė et al., 2019).

#### Biomarkers and Prognosis

Nascent transcription is a promising approach for the study of molecular mechanisms of complex diseases. Chu et al. (2018) developed a novel chromatin run-on and sequencing (ChRO-seq) method to map RNA polymerase in cell or tissue samples and assessed nascent transcription in primary human glioblastoma (GBM, ORPHA:360) brain tumors. In order to identify the exact location of active transcriptional regulatory elements (TREs), the authors developed discriminative regulatory-element detection from GRO-seq, high-definition (dREG-HD), an epsilon-support vector regression (SVR) with a Gaussian kernel, which uses GRO-seq, PRO-seq, or ChRO-seq input data to identify TREs similar to the subset of DNase I hypersensitive sites (DHSs) exhibiting local transcription initiation. Three transcription factors, such as C/EBP, RAR, and NF-kB, whose target genes are correlated with poor clinical outcomes, were identified (Chu et al., 2018).

Epithelial ovarian cancer (EOC) presents a heritable component of 22%. Lu et al. (2018) performed a transcriptome-wide association study (TWAS) among a large cohort (97,898 women) of European ancestry. Expression prediction models, using the elastic net method, were built for protein-coding genes, miRNAs, lncRNAs, processes transcripts, and immunoglobulin and T-cell receptor genes, identifying 35 genes associated with EOC risk, including *FZD4*, which is a potential novel risk locus (Lu et al., 2018). The elastic net method (implemented in the R package “glmnet”) was also used by Yang et al. (2018) to analyze the role of DNA methylation in EOC. The authors used data from the Framingham Heart Study (FHS) Offspring Cohort to generate methylation prediction models for 223,959 CpGs. The prediction models were applied to GWAS data from control and EOC cases, finding 89 CpGs with methylation levels predicted to be associated with EOC risk (Yang et al., 2018).

B-cell precursor acute lymphoblastic leukemia (BCP-ALL, ORPHA: 99860) is responsible for about 80% of all the ALL cases. In order to identify new risk loci for BCP-ALL, Vijayakrishnan et al. (2017) analyzed GWAS data from two different cohorts identifying rs35837782 and rs4762284 risk loci for BCP-ALL, at 10q26.13 and 12q23.1, respectively. The epigenetic profile of association signals at each of the two new risk loci, a multivariate HMM was used to binarize Chip-seq data from GM12878 lymphoblastoid cells inferred from ENCODE Histone Modification data (Vijayakrishnan et al., 2017).

#### Disease Classification

Liu et al. (2019) developed a novel neighborhood-based computational model called NBMDA to infer potential miRNA-disease associations. This model constructs and integrates both disease and integrated miRNA similarity networks based on the disease semantic similarity, miRNA functional similarity, and Gaussian interaction profile kernel similarity for miRNAs and diseases. The k-nearest neighborhood (KNN) method is then applied to the two integrated similarity networks, solving the occurrence of sparse known miRNA-disease associations. The concept of common neighbors is used to calculate potential miRNAs-diseases associations. The authors used esophageal neoplasms (ORPHA:506136), breast neoplasms, and colon neoplasms (ORPHA:100080) as case studies and found 47, 48, and 48, respectively out of the top 50 predicted miRNAs, which were validated by relevant databases or related literature separately, demonstrating the excellent predictive performance of this model and its utility for disease treatment (Liu et al., 2019).

Telomerase reverse transcriptase (TERT), the protein component of telomerase complex is not only involved in aging-related disorders and cancer, but also in RDs, such as aplastic anemia and dyskeratosis congenital. Furthermore, TERT shows non-telomeric functions and could be implicated in the regulation of approximately 300 genes (Hou et al., 2017). Hou et al. (2017) investigated TERT interaction networks using several bioinformatic databases, such as miRDB, which uses the MirTarget tool for miRNA target prediction and functional annotations and GeneMANIA, a fast heuristic algorithm derived from ridge regression that integrates multiple functional association networks and predicts gene function from a single process-specific network using label propagation. The authors found interactions between TERT and PABPC1, SLC7A11 and TP53 genes, indicating a possible role for TERT in RDs, such as Rift Valley Fever (ORPHA:319251) (Hou et al., 2017).

### Combination of Supervised and Unsupervised Algorithms

In this section, we present the manuscripts that referenced both unsupervised and supervised methods/tools for epigenomic analysis.

#### Diagnosis: Mutation Detection and/or Prediction

Less frequent genetic variants are gaining relevance in complex disorders and present a new challenge for genomic research. To investigate how epigenetics can aid aggregate rare-variant association methods (RVAM), Bien et al. (2017) analyzed the location of variants associated with colorectal cancer (CRC, ORPHA:443909). Hierarchical clustering using Pearson correlation as the distance measure and complete linkage followed by the optimal ordering of leaves was used for the categorization of the 127 samples from NIH Roadmap Epigenomics and Encyclopedia of DNA Elements (ENCODE) projects in order to map active regulatory elements (ARE). ChromHMM, a software based on multivariate HMM, was used for the definition of chromatin accessible regions and log-additive logistic regression was used to analyze GWAS data. The authors found that CR ARE were enriched for more significant CRC associations with both common and rare variants (Bien et al., 2017).

Systemic sclerosis (SSc, ORPHA:90291) is a chronic autoimmune disease of unknown etiology with significant clinical heterogeneity and no therapeutic options, leading to high mortality rates. Moreno-Moral et al. (2018) integrated differential expression and expression quantitative trait locus (eQTL) analyses in monocyte-derived macrophages to elucidate the link between macrophage transcriptome and SSc disease variants. Clustering was performed using correlation distance and the method “ward.D” from *hclust* R function while PLINK was used for quality control of the genotype data. This analysis allowed the identification of several *cis*-regulated genes in SSc macrophages, particularly *GSDMA*, which carries an SSc risk variant, regulating the expression of neighboring genes (Moreno-Moral et al., 2018).

The use of genome-wide methylation arrays for identifying epigenetic patterns associated with RDs has increased over the last years. Epigenetic signatures in combination with genomic sequencing can aid diagnosis, the screening of large cohorts, and help find variant significance (Bend et al., 2019). Recently, genes involved in chromatin regulation have been implicated in neurodevelopmental disorders. Bend et al. (2019) performed genome-wide DNA methylation analysis on the peripheral blood of 22 patients with Helsmoortel-van der Aa (ADNP, ORPHA:404448) syndrome. Hierarchical clustering and multiple dimensional scaling allowed for the identification of two distinct episignatures enriched with genes involved in neuronal function. These two episignatures were used to train a multi-class SVM with linear kernel on the training cohort, allowing for the identification of three previously undiagnosed patients with ADNP syndrome from a large cohort (*n* = 1,150) of patients with unresolved developmental delay (Bend et al., 2019).

Vuckovic et al. (2020) performed a genome-wide discovery analysis to investigate 29 blood cell phenotypes from the UK Biobank cohort, plus additional 15 phenotypes from the Blood cell consortium (BCX). A Bayesian method was used for sentinel (a clump tag variant or a trait-specific conditionally independent signal) annotation, while SpliceAI, a state-of-the-art neural net classifier (Jaganathan et al., 2019), was used to predict fine mapped (FM) variants affecting the splicing process. DeltaSVM (Lee et al., 2015), a support-vector machine classifier, was used to predict allele- and cell-specific impact of FM variants in chromatin accessibility. The authors also assessed the validity of the omnigenic model, which states that complex trait heritability is the product of two types of genes (core vs. peripheral) (Vuckovic et al., 2020).

Whole-genome sequencing (WGS) has revealed disease-causing variants undetected by other genetic tests in RDs, particularly the ones located in non-coding regions, namely 5′ and 3′ untranslated regions (UTR), enhancer and promoter regions, and miRNA genes (Smedley et al., 2016). However, the number of regulatory variants related to Mendelian disorders remains low. Smedley et al. (2016) developed Genomiser for the prioritization of NCVs and the discovery of causative SNVs of Mendelian disorders. This framework is divided into two major components: (1) the regulatory Mendelian mutation (ReMM) framework, a machine learning method for scoring NCVs based on SMOTE with several nearest neighbors *k* = 5, and (2) an RF classifier, for ranking NCVs in WGS data. Performance was tested using a 10-fold “cytogenetic band-aware” cross-validation scheme. Genomiser identified the causative regulatory Mendelian mutation as the top candidate out of the 4 million plus variants in a whole genome in 77% of the analyzed samples (Smedley et al., 2016).

#### Biomarkers and Prognosis

Pheochromocytomas and paragangliomas (PCPGs, ORPHA: 29072) are tumors of the adrenal medulla or extra-adrenal paraganglia respectively, with high heritability and no reliable biomarkers. Ghosal et al. (2020) used different tools to identify a prognostic long intervening non-coding RNA (lincRNA) signature associated with metastasis in PCPGs. Four ML models, elastic net, LASSO, Ridge, and CART (classification and regression trees) were used to classify samples into five molecular subtypes of PCPG. This model can be used as a potential diagnostic tool for several molecular subtypes and/or aggressive/metastatic PCPGs (Ghosal et al., 2020).

Wen et al. (2017) combined microarray and RNA data, and clinical information from patients with GBM to study the association between malignant tumor degree and gene methylation level, while logistic regression was used to assess methylated genes associated with the tumor malignant degree of patients. A total of 668, 412, 470, and 620 genes relevant with methylation or demethylation were associated with the malignant degree, Grade1, 2, 3, and 4, respectively of tumor. *CCL11* and *LCN11* were significantly related to GBM progression (Wen et al., 2017).

Hierarchical clustering as well as a supervised analysis using the “signed average expression” survival prediction method were used by Lietz et al. (2020) to test the validity of a set of prognostic signatures (*5-miRNA* and *22-miRNA* profiles) in two osteosarcoma (ORPHA:668) cohorts. Furthermore, the authors observed that sets of experimentally validated gene (mRNA) targets of the prognostic miRNAs presented robust outcome predictive function, suggesting a possibly active miRNA/mRNA network. A composite model integrating information from pathologic necrosis combined with miRNA biomarkers was proposed, allowing improved and refined stratification into three relevant prognostic groups (very favorable, very unfavorable, and intermediate) (Lietz et al., 2020).

Conventional GWAS was performed by Luzón-Toro (2015) in a cohort of sporadic medullar thyroid carcinoma (sMTC, ORPHA:1332) and juvenile papillary thyroid carcinoma (jPTC, ORPHA:146), two rare tumors of the thyroid gland. PLINK was used for GWAS analysis and the multifactor-dimensionality reduction (MDR) method was used to infer possible epistatic interactions between pairs of genes. The authors found two epistatic interactions (interaction of genetic variations at two or more loci to produce a phenotypic outcome) in sMTC and three in jPTC, being lincRNAs among the epistasis found, showing the increasing relevance of these elements in cancer research (Luzón-Toro, 2015).

A group of recurrent or fatal ACC was found to carry a unique CpG island methylator phenotype — CIMP-high. To identify biomarkers specific for this group, Mohan et al. (2019) used data from the Cancer Genome Atlas project on ACC (ACC-TCGA). Logistic regression was used to identify transcripts that are able to predict CIMP-high status. Pheatmap was used for unsupervised complete hierarchical clustering. Through this approach, the gene *G0S2* was identified, hypermethylated, and silenced exclusively in 40% of ACC, representing a hallmark amenable to be assessed using routine molecular diagnosis (Mohan et al., 2019).

#### Disease Etiology and Classification

Integration of DNA-methylation profiles with the somatic genomic landscape was used to propose a three-class classification system for ACC. The authors performed RNA-seq data analysis using a SVM classifier uncovering fusion events in 78 specimens. Unsupervised NMF consensus clustering was used to divide miRNA samples into groups according to similar abundant profiles. Unsupervised consensus clustering of DNA methylation data of the entire cohort was performed using Euclidean distance and PAM. Boruta method was used to calculate the DNA methylation signature of the CIMP tumor group, identifying an optimal signature containing 68 probes representing 59 genes (Zheng et al., 2016).

#### Therapies

Rendeiro et al. (2020) used a multi-omics approach to assess the cell composition and immunophenotype, gene expression, and chromatin accessibility in order to study the regulatory dynamics of ibrutinib treatment in chronic lymphocytic leukemia (CLL, ORPHA:67038). Python library GPy, a variable radial basis function (RBF) kernel, and a constant kernel were used to model the temporal effect of ibrutinib in each cell type as a function of time. The authors also used the “mixture of hierarchical Gaussian process” (MOHGP) method to cluster regulatory elements according to their temporal pattern. The MOHGP class from the GPclust library (GPclust.MOHGP) was used with a Matern52 kernel (GPy.kern.Matern52) and an initial guess of four region clusters. Enrichment of genes associated with regulatory elements was carried out through the Enrichr API for 15 databases of gene sets. Hence, their results demonstrate the value of combined multi-omics profiling for patient-specific treatment monitoring (Rendeiro et al., 2020).

## Challenges and Future Perspectives

The development of new methods for the analysis of epigenomic marks, alongside with the integration of information from genetic and epigenetic profiles as well as other “omics” has expanded our knowledge about the complex nature of RDs. Methods, such as nano-hmC-Seal (for 5-hydroxymethylcytosine analysis) (Han et al., 2016), single chromatin molecular analysis in nanochannels (SCAN — for single DNA and chromatin molecule analysis) (Murphy et al., 2013) and reduced representation bisulfate sequencing (RRBS), a high-throughput technique for genome-wide methylation profile analysis (Hamamoto et al., 2019), already in practice for RDs and common diseases, are improving epigenetic studies, particularly in the case of rare cell populations and limited input DNA. However, according to Kerr et al. (2020), in the RDs field, few studies explore the potential of incorporating epigenomic analysis in combination with other “omics” and the majority of studies analyzing epigenomic information are related to rare cancers, in accordance with our findings. The development and generalization of high-throughput analysis have increased the amount of information to be analyzed, integrated, and processed. AI technologies can automate tasks currently requiring human intervention and have been applied in the analysis of a diverse array of data, contributing to advance disease characterization and classification, diagnosis, and therapy development in RDs (Brasil et al., 2019). Unsupervised AI tools are generally used for data clustering since no label has to be assigned to the data. In this review, 11 articles only reported the use of unsupervised tools, specifically for data clustering (Ammerpohl et al., 2013; Assié et al., 2014; Kaur et al., 2014; Han et al., 2016; Berdasco et al., 2017; Koduru et al., 2017; Rivera-Mulia et al., 2017; Sorenson et al., 2017; Waszak et al., 2018; Nagaraja et al., 2019; Job et al., 2020). The analysis of large-scale methylation arrays is difficult with traditional clustering tools due to the high-dimensional data-analysis problem. Hence, model-based clustering tools, such as RPMM and MOHGP offer better solutions (Houseman et al., 2008). For non-model based approaches, MDR can be used for high-dimensional data analysis (Ritchie et al., 2001).

The analysis of the combination of data obtained from different omics studies, particularly regarding genotype and gene expression is essential for complete disease comprehension, but it is also challenging. Supervised analysis has been restricted to biological problems in which a good balance between variables and data is observed (Esteban-Medina et al., 2019). In this work, most supervised tools have been applied for diagnosis (Mo et al., 2014; Crippa et al., 2014; Fu et al., 2014; Farh et al., 2015; McMaster et al., 2018; Aref-Eshghi et al., 2019; Pranckėnienė et al., 2019; Cochran et al., 2020; Kurkiewicz et al., 2020). In RDs, small sample sizes allied to complex levels of data structure and differences among the characteristics of patients can hinder the application of AI tools. Establishment of research consortiums or collaborations is crucial to obtain larger patient cohorts. This is particularly relevant considering the low number of collaborations among the papers retrieved by this review (Figure 1C). Furthermore, defects in data pre-processing (e.g., removal of outliers), the use of excessively large datasets for algorithm training, and the promiscuous use of the same data instances in both training and testing phases can lead to erroneous results and model overfitting (Chicco, 2017). Cross-validation and regularization can be used to avoid overfitting (Chicco, 2017; Hamamoto et al., 2019). Over-sampling (SMOTE) and a 10-fold cross-validation scheme are employed by Genomiser, a tool used for the diagnosis based on an RF classifier (Smedley et al., 2016). However, the predictive performance of RFs is lower compared to other methods, such as SVM, due to the fact that one incorrect decision affecting one data subset can affect the following sequencing leading to error propagation (Esteban-Medina et al., 2019). SVM has been used in methylation data and RNA-seq analysis for diagnosis (Zheng et al., 2016; Aref-Eshghi et al., 2019; Bend et al., 2019; Vuckovic et al., 2020). Deep learning tools, such as neural networks (NNs) have been applied to computational biology problems with success and present several advantages compared to traditional ML tools, such as the ability to operate directly on a sequence without manual feature extraction. However, NNs need initial weight values for efficient training and have low interpretability (Bousquet et al., 2004; Ehsani-Moghaddam et al., 2018). Convolution neural networks (CNNs) that allow direct training on the DNA sequence, eliminating the need to feature definition, and reducing the number of the parameter in the model are also a good approach to the complex problem of “omics” data integration (Angermueller et al., 2016; Hamamoto et al., 2019). Despite these advantages, no reports of the use of deep leaning tools were described for RDs regarding epigenomics, suggesting that this is an unexplored avenue for the application of particular AI tools.

Ultimately, the choice of the AI tool will depend on the type of data set and biological problem to be solved, keeping in mind that there is no “one size fits all” tool and that for many cases, the solution can be the application of ensemble learning, in which several individual learners are combined to form an individual learner (Dey, 2016).

Our study has some limitations. First, we only searched for Medline database — Pubmed. Although it is the most complete biomedical literature database, insightful data present in other databases may not have been included. Secondly, our keyword search may not have reached all relevant articles, probably due to the absence of the keywords related to AI, ML, and/or epigenetics both in the MeSH terms as in the author-defined keywords. Furthermore, we observed some inespecificity within our search, since many papers retrieved were focused on bioinformatics rather than AI tools. The use of similar statistical tools between these two fields may account for this outcome.

This review explores the potentialities of AI tools applied to epigenetics in the context of RDs and despite the reduced number of studies incorporated, there is an expanding application of such tools to other disorders besides rare cancers. We believe that the dissemination of the tools and approaches already used will foster further application in other RDs that have complex traits and face the “missing heratibility” problem, which impairs discovery, diagnosis, and patients care, such as congenital disorders of Glycosylation.

## Conclusion

The use of AI, particularly ML for epigenetic data analysis, integration, and interpretation is a growing field with the potential to address significant issues concerning RDs. Particularly, the improvement of diagnosis rate, provision of prognostic biomarkers, pathophysiology, and therapy development. The application of strategies already applied in common disorders can expedite the use of AI in epigenetic studies for RDs and many tools are already being applied. Hence, AI applied to the expanding field of epigenetics can help elucidate the involvement of epigenomes in RDs pathophysiology, fostering new diagnostic tools and new therapeutic avenues. However, it is essential to keep in mind that before the generalized application of AI in research and ultimately, in the clinical context for RDs, AI methods as the data being analyzed need to undergo some refinement to avoid erroneous data interpretation.

## Author Contributions

VR conceived and supervised the study. CN performed the literature search and selection, retrieved the manuscript data, and contributed to the manuscript writing. SB performed the literature selection, retrieved the manuscript data, and wrote the manuscript. TR retrieved the manuscript data, reviewed AI data inclusion, and designed the plots. MF retrieved the manuscript data and contributed to the manuscript writing. PV reviewed the manuscript writing and supervised the study. GV performed the literature search, reviewed the AI data inclusion, and reviewed manuscript writing. All authors contributed to the article and approved the submitted version.

## Conflict of Interest

The authors declare that the research was conducted in the absence of any commercial or financial relationships that could be construed as a potential conflict of interest.

## Footnotes

1 About Rare Diseases | www.eurordis.org Available online: https://www.eurordis.org/about-rare-diseases (accessed on Nov 10, 2020).

2 https://notebook.community/mzwiessele/GPclust/notebooks/index

3 http://www.sthda.com/english/articles/36-classification-methods-essentials/149-penalized-logistic-regression-essentials-in-r-ridge-lasso-and-elastic-net/

4 https://www.cancer.gov/publications/dictionaries/cancer-terms/def/somatic-mutation (accessed on December 15, 2020).

5 http://pfam.xfam.org/
