# Supplementary material for: Artificial Intelligence in Epigenetic Studies: Shedding Light on Rare Diseases
Source: Front Mol Biosci. 2021 May 5;8:648012. doi: 10.3389/fmolb.2021.648012 (PMC8131862; doi:10.3389/fmolb.2021.648012)
Supplement: Supplementary file 1 [file Data_Sheet_1.docx]

Supplementary Material

Supplementary Note S1.

**S1. Python programming language script – 1**

In this literature review, we used the script (Python 3.7.3) below to select the papers and retrieve the correspondent Medline data (Title, Abstract, etc). We ran it in a Linux operating system. To run the script, the user should replace the information in the variables: base_path; Entrez.email, and Entrez.api_key (highlighted in grey) by your information, and use the input file called “keywordsbulk.xls", with the four lists of keywords listed on Table S1. The output file of this script is called “papers.dat”.

import pandas as pd

import numpy as np

import pickle

from Bio import Entrez, Medline

import time

base_path = “/home/johndoe/projects/p_epigenomic_ai_rd/"

file_loc = base_path + "keywordsbulkmine.xls"

df = pd.read_excel(file_loc, na_values=['NA'], usecols = "A")

df=df.dropna()

rd_g = df['RD_G'].tolist()

#print(rd_g)

df = pd.read_excel(file_loc, na_values=['NA'], usecols = "B")

df=df.dropna()

ai_g = df['AI_G'].tolist()

#print(ai_g)

df = pd.read_excel(file_loc, na_values=['NA'], usecols = "C")

df=df.dropna()

epi_g = df['Epi_G'].tolist()

#print(epi_g)

df = pd.read_excel(file_loc, na_values=['NA'], usecols = "D")

df=df.dropna()

t_g = df['T_G'].tolist()

#print(t_g)

quadrupleterms = []

for i in rd_g:

for j in ai_g:

for k in epi_g:

for l in t_g:

aterm = i.lower() + ' ' + j.lower() + ' ' + k.lower() + ' ' + l.lower()

quadrupleterms.append(aterm)

#print(quadrupleterms[0:3])

tripleterms = []

for i in rd_g:

for j in ai_g:

for k in epi_g:

aterm = i.lower() + ' ' + j.lower() + ' ' + k.lower()

tripleterms.append(aterm)

#print(tripleterms[0:3])

allterms = quadrupleterms + tripleterms

#print(allterms)

print("############################# Finish Part1 ################################")

Entrez.email = "johndoe@mail.com"

Entrez.api_key="API_KEY_identifying_John_to_NCBI servers"

allpmids = []

for ttt in allterms:

handle = Entrez.esearch(db='pubmed', sort='relevance', retmax='1000', term=ttt, usehistory="n")

pmids = Entrez.read(handle)['IdList']

#pmids = Entrez.read(handle)

allpmids.extend(pmids)

print(len(allpmids))

print("############################# Finish Part2 ################################")

allpmids = list(set(allpmids))

out_handle = open("auxiliary.txt", "w")

allpmidsaux = ','.join(allpmids)

fetch_handle = Entrez.efetch(db='pubmed', rettype='medline', retmode='text', id=allpmidsaux)

data = fetch_handle.read()

fetch_handle.close()

out_handle.write(data)

out_handle.close()

with open("auxiliary.txt") as auxilhandle:

therecords = Medline.parse(auxilhandle)

recordslst = list(therecords)

auxilhandle.close()

with open('papers.dat','wb') as filename:

pickle.dump(recordslst, filename)

print("############################# Finish Part3 ################################")

Table S1 - List of keywords used by the script (Python 3.7.3) to create the triple and quadruple search terms presented in Supplementary Figure 1. This list should be included in an excel file called “keywordsbulk.xls" in order to be used by the script.

| RD_G | **AI_G** | **Epi_G** | **T_G** |
| --- | --- | --- | --- |
| Rare Disease | Artificial Intelligence | Epigenome | Data browser |
| Rare Disorder | Computational intelligence | Epigenomic | Rpository |
| Congenital Disorders of Glycosylation | Machine intelligence | Epigenetic | Database |
| CDG | Computer reasoning | Episignature | Tool |
| Rare metabolic disorder | Computer assisted learning | Epivariation | Approach |
| Rare inherited metabolic disease | Machine learning | Epimutation | Pipeline |
|  | Deep learning | Methylome | Method |
|  | Deep neural network | Methylomic | Software |
|  | Big data | DNA methylation | Data analysis |
|  | Data mining | Histone modification | Integrative analysis |
|  | Bioinformatic | Noncoding RNA | Script |
|  | Statistical learning | Non-coding RNA |  |
|  | Computational biology | ncRNA |  |
|  |  | Long non-coding RNA |  |
|  |  | Long ncRNA |  |
|  |  | lncRNA |  |
|  |  | Short non-coding RNA |  |
|  |  | Short ncRNA |  |
|  |  | sncRNA |  |
|  |  | microRNA |  |
|  |  | miRNA |  |
|  |  | Short interfering RNA |  |
|  |  | siRNA |  |
|  |  | piwi-interacting RNA |  |
|  |  | piRNA |  |
|  |  | Chromatin |  |
|  |  | Chromatin remodeling |  |
|  |  | Chromatin accessibility |  |

**S2. Python programming language script - 2**

In this literature review, we used the script (Python 3.7.3) below to extracts information to latex from “papers.dat” (output script - 1) and to produce a pdf to each article with that information: title, authors, date, abstract, mesh terms, Source, PubMed Unique, Identifier, PubMed Central Identifier. We ran it in a Linux operating system (output script - 2). To run the script, the user should replace the path (highlighted in grey) by the directory where saved the file “papers.dat”.

import os

from pylatex import Document, Section, Subsection, Command, LargeText

from pylatex.utils import italic, NoEscape, bold

#from Bio import Entrez, Medline

# import bio

import pickle

os.chdir(os.path.dirname(“/home/johndoe/projects/p_epigenomic_ai_rd/"))

with open("papers.dat", "rb") as fp: # Unpickling

papers = pickle.load(fp)

i = 0

for p in papers:

doc = Document()

doc.preamble.append(Command('title',p['TI']))

doc.preamble.append(Command('author','; '.join(p['FAU'])))

doc.preamble.append(Command('date',p['EDAT']))

#doc.append(NoEscape(r'\maketitle'))

with doc.create(Section('A second section')):

doc.append(NoEscape(r'\maketitle'))

doc.append(LargeText(bold('Abstract\n\n')))

doc.append('\n')

doc.append(p['AB'])

doc.append('\n\n\n\n')

doc.append('Mesh Terms\n')

if 'MH' in p:

doc.append(p['MH'])

doc.append('\n\n')

doc.append(p['SO'])

doc.append('\n\n')

doc.append('PMID: ' + p['PMID'])

doc.append('\n\n')

if 'PMC' in p:

doc.append('PMC: ' + p['PMC'])

doc.generate_pdf(str(i+1), clean_tex=True)

i = i+1

print(i)

#if i > 0:

# break

**Supplementary Figure 1 –** List of all keywords and search combinations used for this literature review.


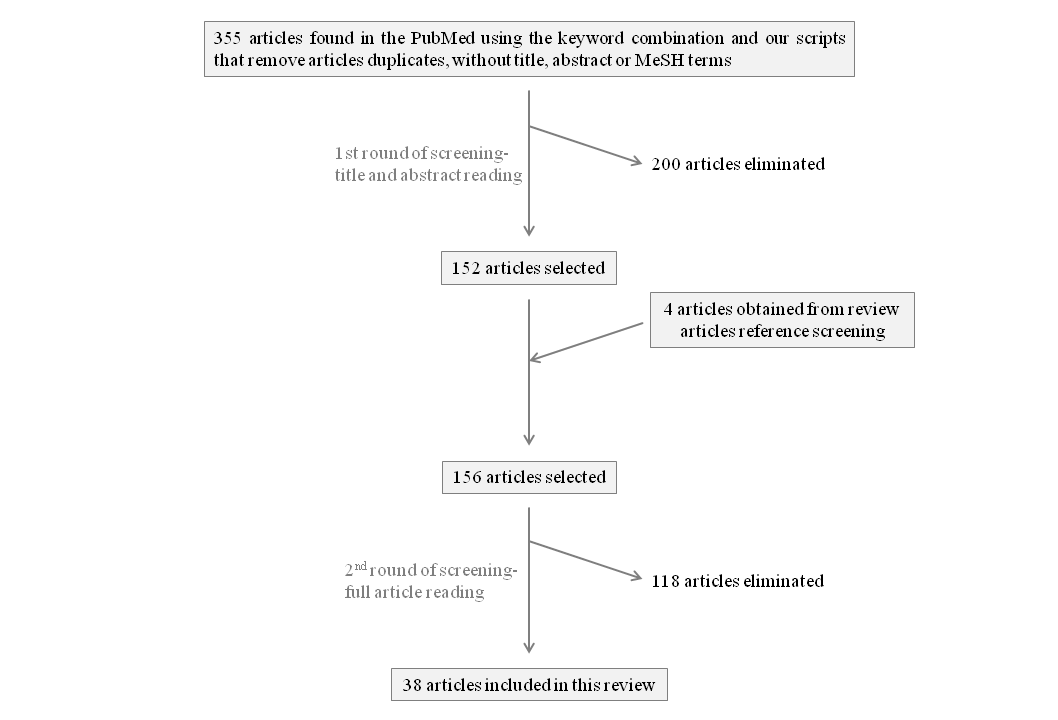


**Supplementary Figure 2.** Diagram of the inclusion/elimination process used for manuscript selection.
